# Enhancement of Motor Cortical Gamma Oscillations and Sniffing Activity by Medial Forebrain Bundle Stimulation Precedes Locomotion

**DOI:** 10.1523/ENEURO.0521-21.2022

**Published:** 2022-07-14

**Authors:** Airi Yoshimoto, Yusuke Shibata, Mikuru Kudara, Yuji Ikegaya, Nobuyoshi Matsumoto

**Affiliations:** 1 Graduate School of Pharmaceutical Sciences, The University of Tokyo, Tokyo 113-0033, Japan; 2 Institute for AI and Beyond, The University of Tokyo, Tokyo 113-0033, Japan; 3 Center for Information and Neural Networks, National Institute of Information and Communications Technology, Suita City, Osaka 565-0871, Japan

**Keywords:** gamma, locomotion, medial forebrain bundle, motor cortex, olfactory bulb, sniffing

## Abstract

The medial forebrain bundle (MFB) is a white matter pathway that traverses through mesolimbic structures and includes dopaminergic neural fibers ascending from the ventral tegmental area (VTA). Since dopaminergic signals represent hedonic responses, electrical stimulation of the MFB in animals has been used as a neural reward for operant and spatial tasks. MFB stimulation strongly motivates animals to rapidly learn to perform a variety of behavioral tasks to obtain a reward. Although the MFB is known to connect various brain regions and MFB stimulation dynamically modulates animal behavior, how central and peripheral functions are affected by MFB stimulation per se is poorly understood. To address this question, we simultaneously recorded electrocorticograms (ECoGs) in the primary motor cortex (M1), primary somatosensory cortex (S1), and olfactory bulb (OB) of behaving rats while electrically stimulating the MFB. We found that MFB stimulation increased the locomotor activity of rats. Spectral analysis confirmed that immediately after MFB stimulation, sniffing activity was facilitated and the power of gamma oscillations in the M1 was increased. After sniffing activity and motor cortical gamma oscillations were facilitated, animals started to move. These results provide insight into the importance of sniffing activity and cortical gamma oscillations for motor execution and learning facilitated by MFB stimulation.

## Visual Abstract

**Figure:**
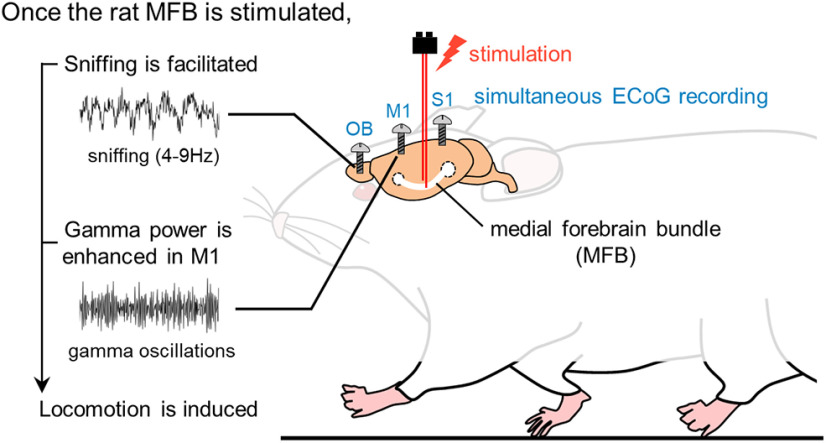

## Significance Statement

Electrical stimulation of the medial forebrain bundle (MFB) in the brain reward system motivates animals to perform a variety of behavioral tasks. However, how MFB stimulation per se influences neural activity and relevant behavior remains incompletely understood. We recorded neural activity from the olfactory bulb (OB), the primary motor cortex (M1), and the primary somatosensory cortex (S1) of freely moving rats and monitored their behavior while regularly stimulating the MFB of the rats. We found that stimulation of the rat MFB facilitated sniffing activity and enhanced gamma oscillations only in the M1, and subsequently induced locomotion. Our findings suggest the possible contribution of gamma oscillations to motor execution and learning facilitated by MFB stimulation.

## Introduction

The medial forebrain bundle (MFB) is a neural fiber tract in rats and humans that connects and passes through various brain regions of the reward system, including the ventral tegmental area (VTA), nucleus accumbens, lateral/medial hypothalamus, sublenticular regions, lateral/medial preoptic regions, diagonal band, and septal area (Nieuwenhuys et al., 1982; Veening et al., 1982; Zahm, 2006; Coenen et al., 2012; Gálvez et al., 2015). A principal component of the MFB is a mesolimbic pathway, a collection of fibers that ascend from dopaminergic neurons in the VTA and terminate in the nucleus accumbens and medial prefrontal cortex (Fenoy et al., 2022). Psychologically, the MFB is considered to serve as the neural substrate for motivation and pleasure, and thus, stimulation of the MFB and surrounding regions has been behaviorally used as a neural and “virtual” reward (Olds and Milner, 1954; Margules and Olds, 1962; Beninger et al., 1977). MFB stimulation ignites hedonic feelings and elicits pleasant bodily sensations in animals, thus highly motivating them to perform a variety of operant and spatial tasks (Carlezon and Chartoff, 2007; Lee et al., 2010; Sun et al., 2012; Farakhor et al., 2019; Kong et al., 2019). Electrical stimulation of the reward system, including the MFB, has also allowed for (tele)control of the spatial navigation of rodents and birds (Talwar et al., 2002; Sun et al., 2012; Huai et al., 2016; Khajei et al., 2019).

Although anatomic and behavioral evidence has led to the assumption that central and peripheral activity is modified by MFB stimulation, little is known about how this activity is indeed affected by MFB stimulation. As both operant conditioning and spatial navigation are accompanied by motor execution, we hypothesized that neural activity in the primary motor cortex (M1) would be modulated by MFB stimulation, which is supported by a previous immunohistochemical study (Hosp et al., 2011). Moreover, because locomotion and breathing have been postulated to be closely correlated, a phenomenon called locomotor-respiratory coupling (Bramble and Carrier, 1983; Potts et al., 2005), we also examined respiratory activity as an index of peripheral function.

To this end, we set out to chronically implant recording electrodes into the M1, primary somatosensory cortex (S1), and olfactory bulb (OB) and insert a stimulation electrode into the MFB (i.e., MFB group) or a neighboring region (i.e., sham group) of rats. We then simultaneously recorded electrocorticograms (ECoGs) in the M1, S1, and OB of freely moving rats while the MFB was repeatedly and regularly stimulated.

## Materials and Methods

### Ethical approval

Animal experiments were performed with the approval of the Animal Experiment Ethics Committee at the University of Tokyo (approval number P29-7) and according to the University of Tokyo guidelines for the care and use of laboratory animals. These experimental protocols were conducted in accordance with the Fundamental Guidelines for the Proper Conduct of Animal Experiments and Related Activities in Academic Research Institutions (Ministry of Education, Culture, Sports, Science and Technology, Notice No. 71 of 2006), the Standards for Breeding and Housing of and Pain Alleviation for Experimental Animals (Ministry of the Environment, Notice No. 88 of 2006) and the Guidelines on the Method of Animal Disposal (Prime Minister’s Office, Notice No. 40 of 1995). All efforts were made to minimize animal suffering.

### Animals

A total of 20 male 8- to 10-week-old Wistar rats (Japan SLC) with a preoperative weight of 180–300 g were individually housed under conditions of controlled temperature and humidity (22 ± 1°C; 55 ± 5%) and maintained on a 12/12 h light/dark cycle (lights off from 7 A.M. to 7 P.M.) with *ad libitum* access to food and water. Rats were habituated to an experimenter via daily handling before experiments were conducted.

### Electrodes

A recording interface assembly was prepared as previously described (Okada et al., 2016; Sasaki et al., 2017; Shikano et al., 2018; Yoshimoto et al., 2021b). In short, the assembly was composed of an electrical interface board (EIB; EIB‐36‐PTB, Neuralynx) and custom-made shell and core bodies created by three-dimensional (3-D) printers. The EIB had a sequence of metal holes for connections with wire electrodes. A particular individual hole was conductively connected with one end of the insulated wire (∼5 cm) using attachment pins, whereas the opposite end was soldered to a corresponding individual electrode during surgery.

Bipolar stimulating electrodes were made from pairs of stainless-steel insulated wires (TOG217-049c, Unique Medical). The distal end of the stimulation electrode was soldered to a two-pin connector protected by epoxy glue to prepare a stimulating electrode assembly (Shibata et al., 2022).

### Surgery

General anesthesia in the rats was induced and maintained with 2–3% and 1–2% isoflurane gas, respectively, with careful inspection of the animal’s condition during the whole surgical procedure. Veterinary ointment was applied to the rat’s eyes to prevent drying. The skin was sterilized with povidone iodine and 70% ethanol whenever we made an incision.

**Figure 1.**
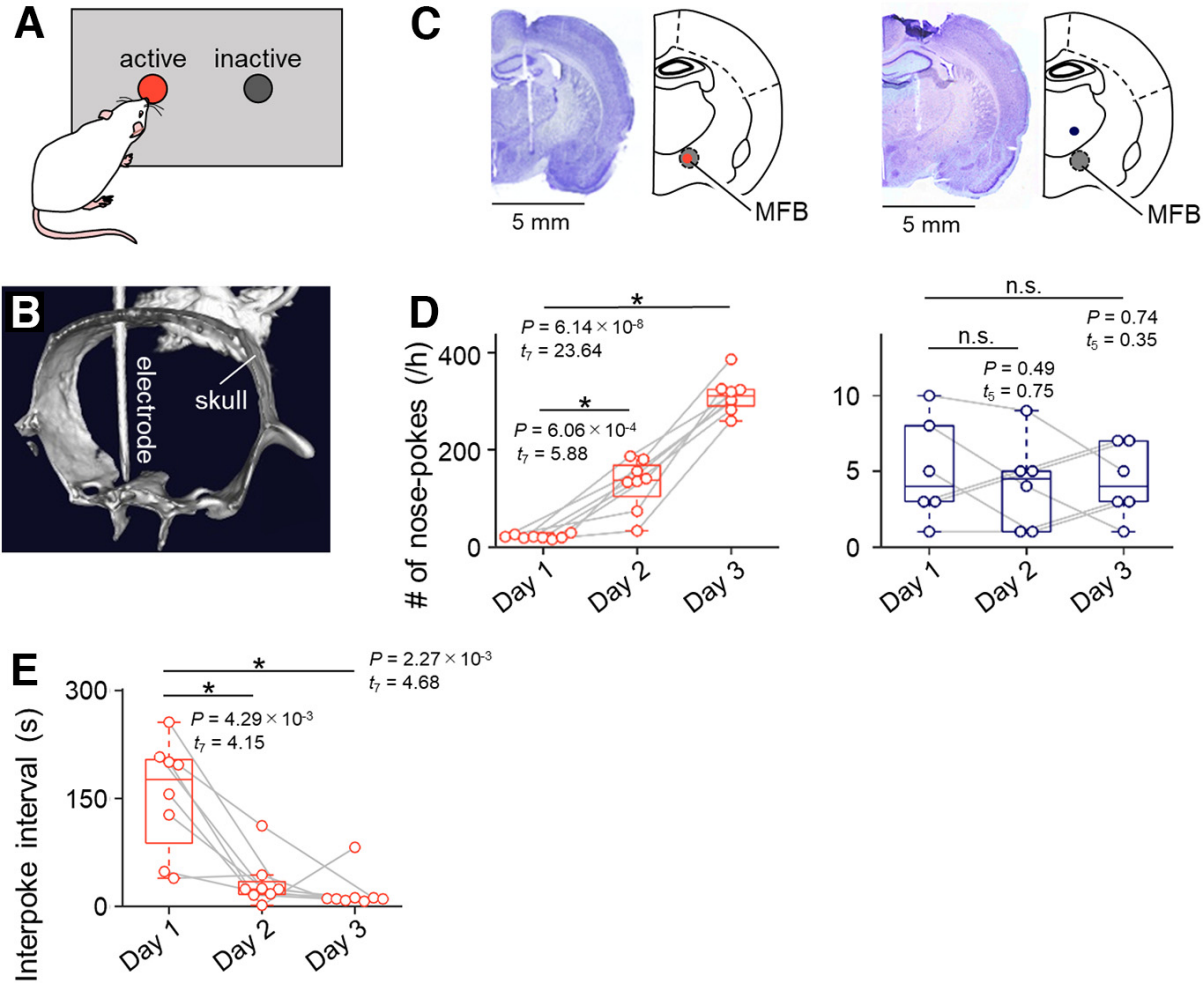
Rats learn to perform a nose-poke test with MFB stimulation. ***A***, A diagram of the experimental setup for the rat nose-poke test. ***B***, A 3D skeletal reconstruction of a rat in which an electrode had been implanted. ***C***, Left, Representative Nissl-stained section image showing the track of the stimulation electrode into the MFB (gray). The tip of the electrode is indicated by a red circle in the simplified brain atlas. Right, The same as left, but for the electrode into a region (indigo) outside of the MFB (gray). ***D***, Left, The number of nose-pokes increased daily in rats in the MFB group (red). Right, The same as left, but for rats in the sham group (indigo). ***E,*** The intervals of nose-pokes decreased daily in rats in the MFB group (red). The *p* and *t* values were obtained by paired *t* tests (*n *=* *8 and 6 rats in the MFB and sham groups, respectively). MFB, medial forebrain bundle.

After anesthesia, electrodes for electromyograms (EMGs) were implanted as previously described (Yoshimoto et al., 2021a). Briefly, a rat was mounted onto a stereotaxic apparatus (SR-6R-HT, Narishige). One wire electrode (AS633, Cooner Wire) was implanted into the trapezius to record EMGs. The scalp was then removed with a surgical knife. A circular craniotomy with a diameter of ∼0.9 mm was performed using a high-speed dental drill. Epidural stainless-steel screws (1.4 mm in diameter, 3 mm in length) were used to record electrocorticograms (ECoGs) from S1 and M1, whereas a smaller screw electrode (1.0 mm in diameter, 4 mm in length) was used to record ECoGs from the OB. The three screw electrodes were stereotaxically implanted into the S1 (2.1 mm posterior and 2.8 mm lateral to bregma), M1 (3.2 mm anterior and 3.0 mm lateral to bregma), and OB (10.0 mm anterior and 1.0 mm lateral to bregma; Yamashiro et al., 2020). In addition, another two stainless-steel screws were implanted into the bone above the cerebellum (9.6 mm posterior and 1.0 mm bilateral to bregma) to serve as ground and reference electrodes. Each of the open edges of the electrodes was soldered to the corresponding open edge of the insulated wires of the recording interface assembly. The bipolar stimulation electrodes (described in the previous section) were stereotaxically implanted unilaterally into the MFB (2.0 mm posterior and 2.0 mm lateral to bregma, and 7.8 mm below the cortical surface; “MFB group” or “MFB-novel group”) or other regions (2.0 mm posterior and 2.0 mm lateral to bregma, and 5.0 mm below the cortical surface; “sham group”). The electrodes were secured to the skull using dental cement. Immediately after implantation, the rat was scanned, and 3-D images were reconstructed by an X-ray microcomputed tomography system (CosmoScan GXII, Rigaku). The parameters for X-ray tomography were as follows: tube voltage, 90 kV; tube current, 88 μA; absorbed dose, 106 mGy; field of view (FOV), 45 mm; voxel size, 90 μm (isotropic); and scan time, 2 min. The electrode placement was roughly located using the reconstructed images (Fig. 1*B*). Rats that did not have the stimulation electrode implanted in the target region were not tested in the following experiments.

Following surgery, each rat was allowed to recover from anesthesia and was individually housed with free access to water and food. For the first 2 d after surgery, the condition of the animals was carefully checked every 3 h except during the night (i.e., 8 P.M. to 8 A.M.). The animals were rehabituated to the experimenter by handling.

While our experimental protocols mandate the humane killing of animals if they exhibit any signs of pain, prominent lethargy, or discomfort, we did not observe such symptoms in any of the 20 rats used in this study.

### Apparatus

An operant chamber (OP-3501, O’hara) with two nose-poke holes (20 mm in diameter) in a soundproof box was used for behavioral tests (described below). The box measured 40 cm in width, 30 cm in depth, and 40 cm in height. Nose pokes were detected by a photoelectric sensor in a hole and recorded using Arduino; note that only one “active” hole was connected to the sensor, whereas the other was not. During electrophysiological experiments, the nose-poke holes were closed (described below).

### Behavioral test

After full recovery from surgery, the animals were habituated to the apparatus for at least 2 d. Following familiarization with the apparatus, rats in the MFB and sham groups performed a nose-poke test for 3 d. In contrast, rats in the MFB-novel group never performed the nose-poke test or underwent familiarization with the apparatus before electrophysiological recordings (also see the next section). The stimulating electrode assembly was attached to a two-core cable. The cable was further connected to an isolator (A365, World Precision Instruments; WPI) and a stimulator (A310, WPI).

Rectangular symmetrical biphasic electric currents were generated by the stimulator. Parameters for the electric currents were as follows: amplitude (for each of the positive and negative phases), 180–300 μA; phase duration (for each phase), 1.0 ms; interphase interval, 0 s; interpulse interval (time between onsets of a positive phase and the next), 100 ms (i.e., pulse frequency, 10 Hz); and burst duration, 500 ms. These stimulations were delivered to the MFB of the rats every 5 s for 3 min.

### *In vivo* electrophysiology

Two days after the behavioral test, rats in the MFB and sham groups underwent electrophysiological recordings. Each rat in the MFB, sham, and MFB-novel groups was allowed to freely explore the operant chamber with its nose-poke holes shut for electrophysiological recordings; note that rats in the MFB-novel group had not been exposed to the operant chamber before the electrophysiological experiments were performed. The stimulating electrode assembly was attached to a two-core cable and connected to an isolator and a stimulator as described in the previous section.

The EIB of the recording interface assembly was connected to a digital headstage (CerePlex M, Blackrock Microsystems), and the digitized signals were amplified and transferred to a data acquisition system (CerePlex Direct, Blackrock Microsystems; Okada et al., 2016; Kuga et al., 2019). ECoG signals were digitized at a sampling rate of 2 kHz.

On the recording day, ECoGs were first recorded for 3 min without any electrical stimulation (i.e., “baseline session”). After the baseline session, ECoGs were recorded for 3 min again, while electrical stimulation was delivered every five seconds (i.e., “stim session”). The parameters of the electric currents were the same as those used in the behavioral test. For analysis, the stim session was split into “prestimulation,” “poststimulation,” and other periods (described below).

### Histology

After the recordings, the rats were anesthetized with an overdose of isoflurane gas and transcardially perfused with 0.01 m PBS (pH 7.4) and 4% paraformaldehyde (PFA) in 0.01 m PBS, followed by decapitation. The brains were soaked overnight in 4% PFA for postfixation and coronally sectioned at a thickness of 100 μm using a vibratome (DTK-1000N, Dosaka EM). Serial slices were mounted on glass slides and processed for cresyl violet staining. To achieve cresyl violet staining, the slices were rinsed in water, ethanol, and xylene; counterstained with cresyl violet; and coverslipped with a mounting agent. The positions of all electrodes were confirmed by identifying dents on the neocortical superficial layer or tracks in the subcortical region in the histologic tissue. Data were excluded from the subsequent analysis if the electrode position was outside the target brain region. Cresyl violet-stained images were acquired using a phase-contrast microscope (BZ-X710, Keyence).

### Data analysis

All data analyses were performed using custom-made MATLAB routines (MathWorks). The summarized values are reported as mean ± SEM. The significance level was set at 0.05, and the null hypothesis was statistically rejected when *p *<* *0.05, unless otherwise specified. For comparison of the power of rhythmic activity in a specific frequency range (see below), common logarithms of the power were taken based on the concept of decibels in the electrophysiological field (Ray et al., 2013; Nakazono et al., 2019; Dubey and Ray, 2020; Reddy et al., 2021); more specifically, the subtraction of logarithms of given raw values practically equals the division of the raw values. Before pairwise comparisons were performed, normality of the sample dataset (calculated by subtraction between corresponding two values) was evaluated by the Shapiro–Wilk test, which tests the null hypothesis that the dataset is drawn from a normally distributed population (Shapiro and Wilk, 1965). If the null hypothesis was rejected, nonparametric tests (i.e., the Wilcoxon signed-rank test) were used for the pairwise comparisons; otherwise, parametric tests (i.e., the paired *t* test) were performed. When multiple pairwise comparisons were required, the significance level was adjusted in accordance with the Bonferroni correction (Fig. 1*D*,*E*). The effect size was evaluated by Cohen’s *d* to find the most effective parameters as needed (Cohen, 1988; Kline, 2004). Sample sizes were not predetermined using statistical methods, but the sample sizes used here were similar to those reported in the field for similar electrophysiological experiments (Konno et al., 2021; Yoshimoto et al., 2021b).

**Figure 2.**
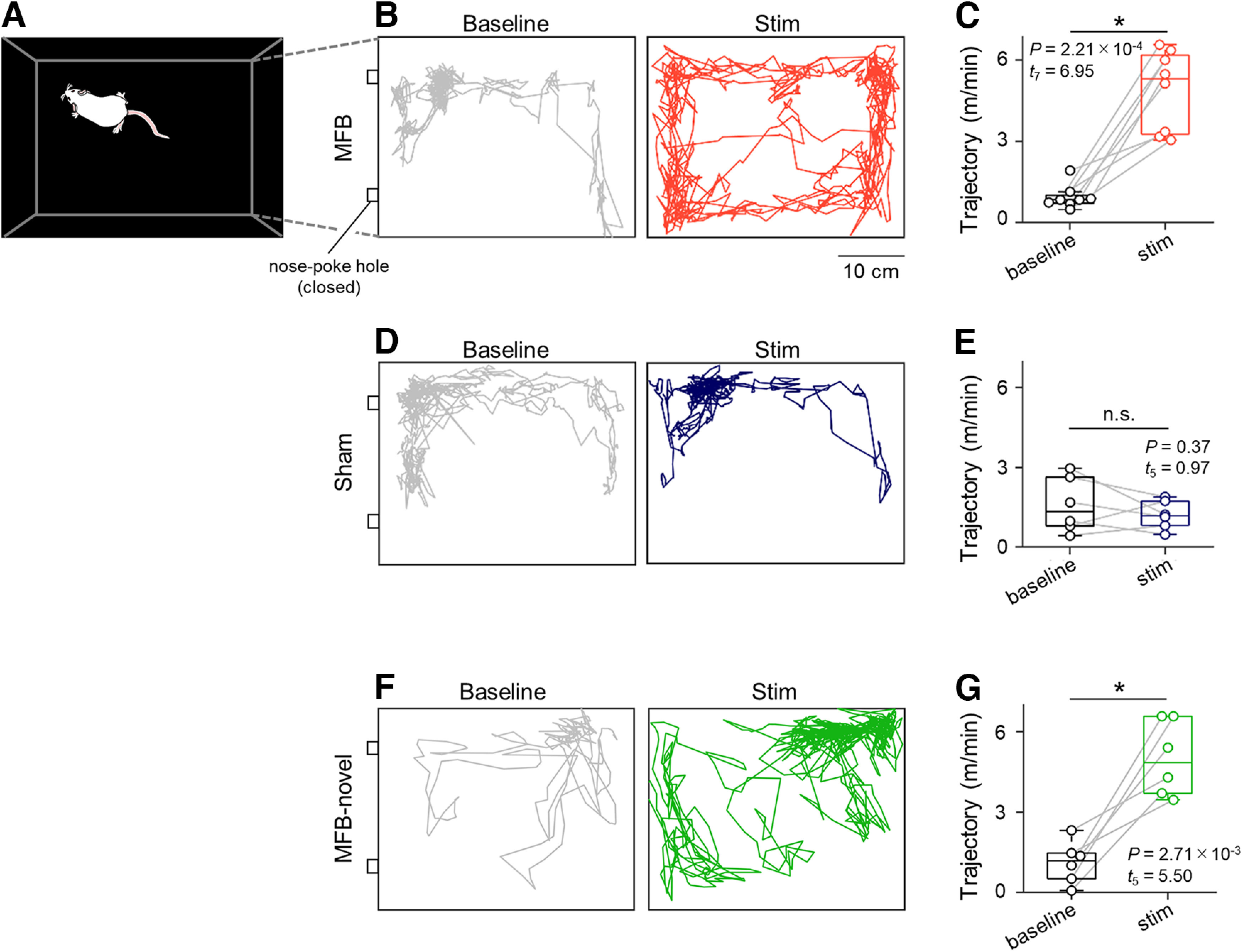
MFB stimulation elicits locomotion. ***A***, Top-view diagram of an open field. ***B***, Representative trajectories of rats in the MFB group during the baseline (left, gray) and stim (right, red) sessions. Both nose-poke holes were shut. ***C***, The distance traveled by rats in the MFB group during the baseline (gray) and stim (red) sessions. ***D***, The same as ***B***, but for rats in the sham group during the baseline (left, gray) and stim (right, indigo) sessions. ***E***, The same as ***C***, but for rats in the sham group. ***F***, The same as ***B***, but for rats in the MFB-novel group during the baseline (left, gray) and stim (right, green) sessions. ***G***, The same as ***C***, but for rats in the MFB-novel group. The *p* and *t* values were obtained by paired *t* tests (*n *=* *8, 6, and 6 rats in the MFB, sham, and MFB-novel groups, respectively). MFB, medial forebrain bundle.

The rats’ behavior was monitored using a web camera operating at 30 fps throughout the experiment. The frame rate of the video was then downsampled to 6 fps. The downsampled data were used to manually mark the rats’ moment-to-moment positions and to track the paths using ImageJ software (National Institutes of Health). The paths traveled by rats were quantified based on the *x* and *y* coordinates of the rats’ heads (Fig. 2*B–G*).

**Figure 3.**
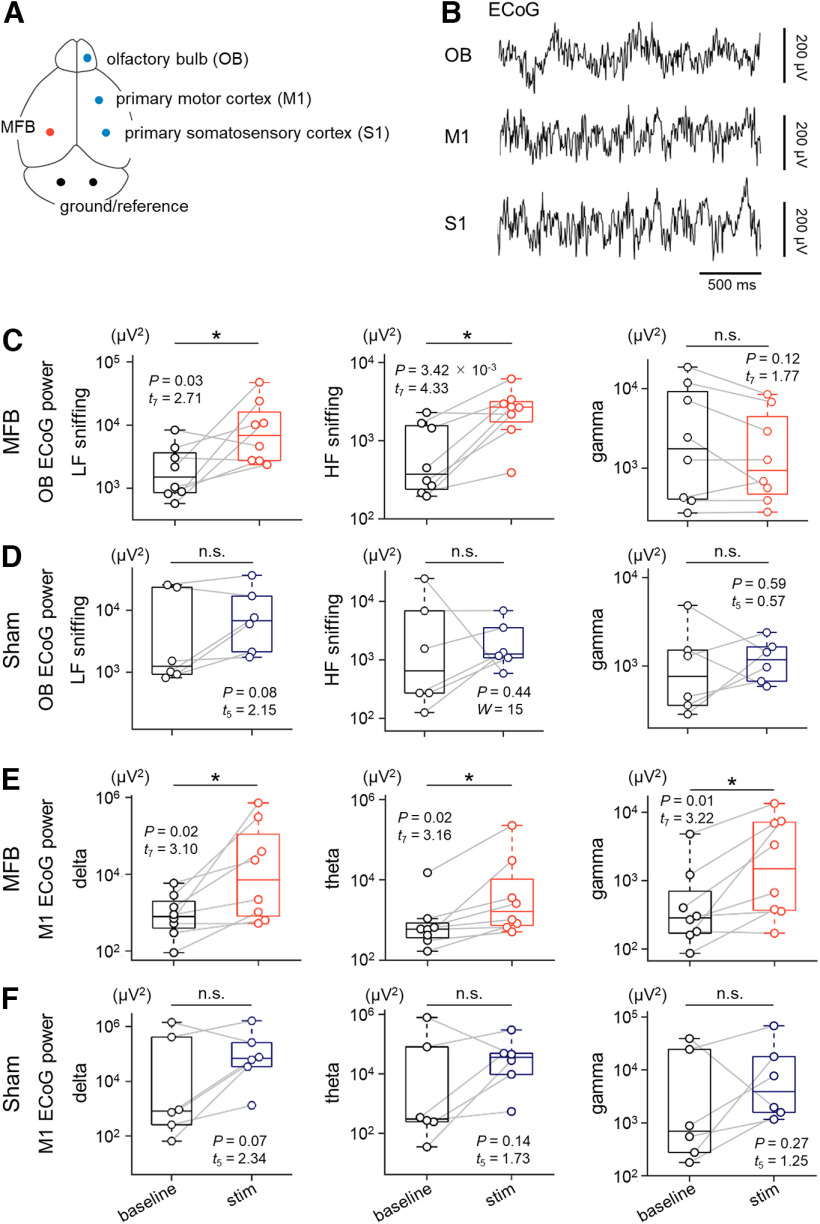
MFB stimulation enhances sniffing components in the OB ECoGs and gamma power in the M1 ECoGs. ***A***, Top-view diagram of the ECoG recording sites (OB, M1, and S1; blue), a stimulation site (MFB, red), and ground/reference sites (black). ***B***, Representative traces of ECoGs in the OB (top), M1 (middle), and S1 (bottom). ***C***, Power of OB ECoGs bandpass-filtered within 1–4 Hz (low-frequency sniffing, left), 4–9 Hz (high-frequency sniffing, middle), and 30–90 Hz (gamma, right) during the baseline (black) and stim (red) sessions in rats in the MFB group. ***D***, The same as ***C***, but for the baseline (black) and stim (indigo) sessions for rats in the sham group. ***E***, Power of M1 ECoGs bandpass-filtered within 0.3–4 Hz (delta, left), 4–8 Hz (theta, middle), and 30–90 Hz (gamma, right) during the baseline (black) and stim (red) sessions for rats in the MFB group. ***F***, The same as ***E***, but for rats in the sham group. The *p* and *t* values were obtained by paired *t* tests or Wilcoxon signed-rank tests (*n *=* *8 and 6 rats in the MFB and sham groups, respectively). MFB, medial forebrain bundle; ECoG, electrocorticogram; OB, olfactory bulb; M1, primary motor cortex; S1, primary somatosensory cortex; LF, low-frequency; HF, high-frequency.

**Figure 4.**
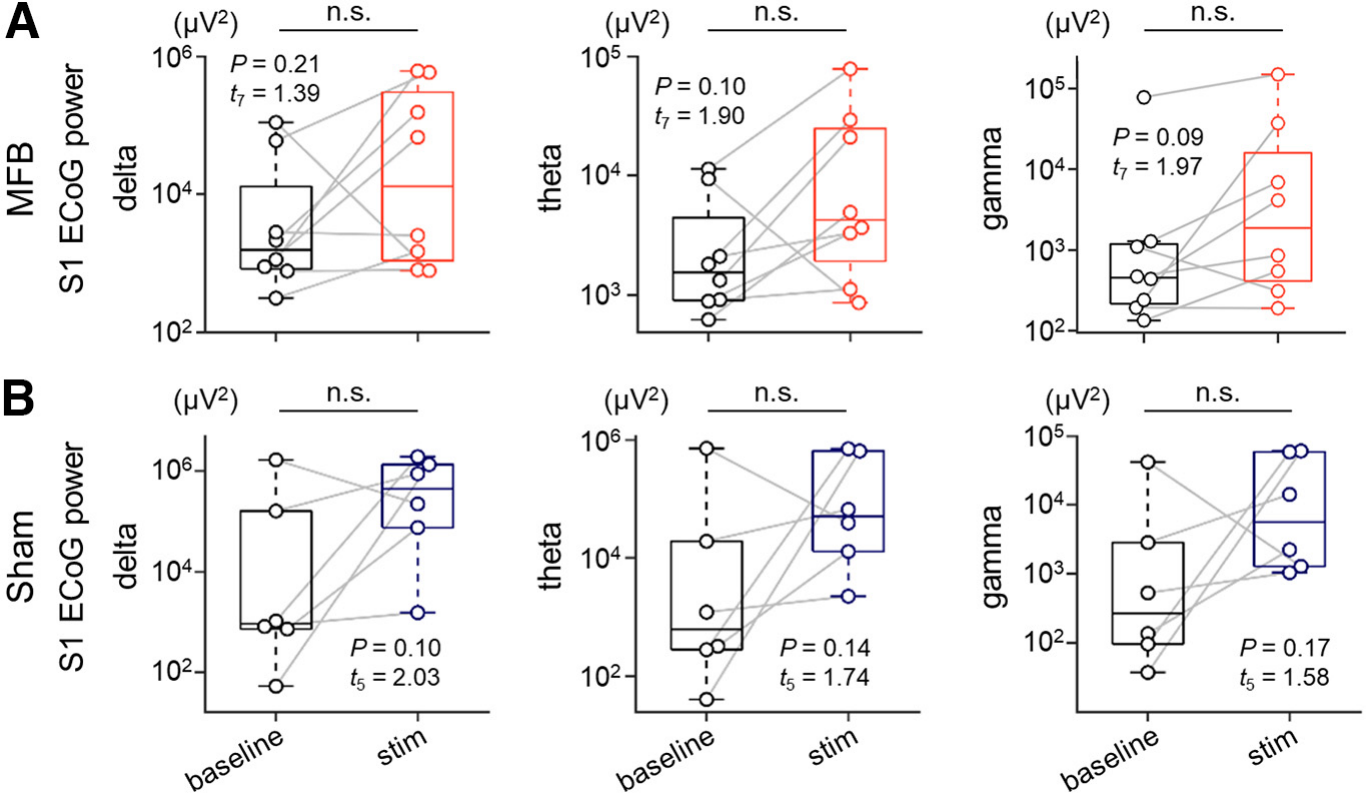
MFB stimulation does not have any effect on S1 ECoG signals. ***A***, Power of S1 ECoG signals bandpass-filtered within 0.3–4 Hz (delta, left), 4–8 Hz (theta, middle), and 30–90 Hz (gamma, right) during the baseline (black) and stim (red) sessions for rats in the MFB group. ***B***, The same as ***A***, but for rats in the sham group. The *p* and *t* values were obtained by paired *t* tests (*n *=* *8 and 6 rats in the MFB and sham groups, respectively). MFB, medial forebrain bundle; ECoG, electrocorticogram; S1, primary somatosensory cortex.

**Figure 5.**
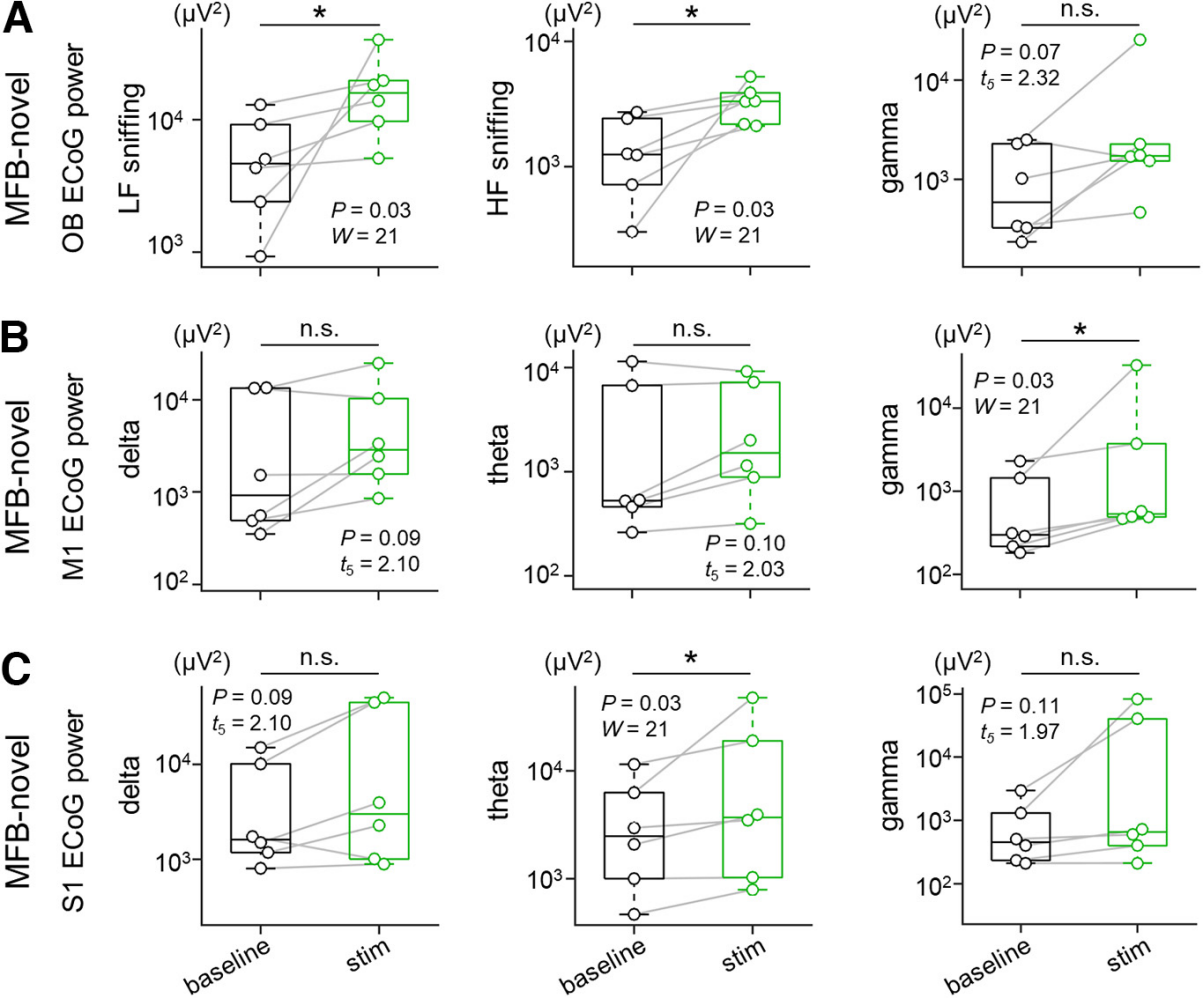
MFB stimulation in a novel environment enhances sniffing components in the OB ECoGs and gamma power in the M1 ECoG signals. ***A***, Power of OB ECoG signals bandpass-filtered within 1–4 Hz (low-frequency sniffing, left), 4–9 Hz (high-frequency sniffing, middle), and 30–90 Hz (gamma, right) during the baseline (black) and stim (green) sessions for rats in the MFB-novel group. Note that rats in the MFB-novel group did not perform the nose-poke test or undergo familiarization with the apparatus before electrophysiology. ***B***, Power of M1 ECoG signals bandpass-filtered within 0.3–4 Hz (delta, left), 4–8 Hz (theta, middle), and 30–90 Hz (gamma, right) during the baseline (black) and stim (green) sessions for rats in the MFB-novel group. ***C***, The same as ***B***, but for the S1 ECoG signals. The *p* and *t* values were obtained by paired *t* tests or Wilcoxon signed-rank tests (*n *=* *6 rats in the MFB-novel group). MFB, medial forebrain bundle; ECoG, electrocorticogram; OB, olfactory bulb; M1, primary motor cortex; S1, primary somatosensory cortex; LF, low-frequency; HF, high-frequency.

**Figure 6.**
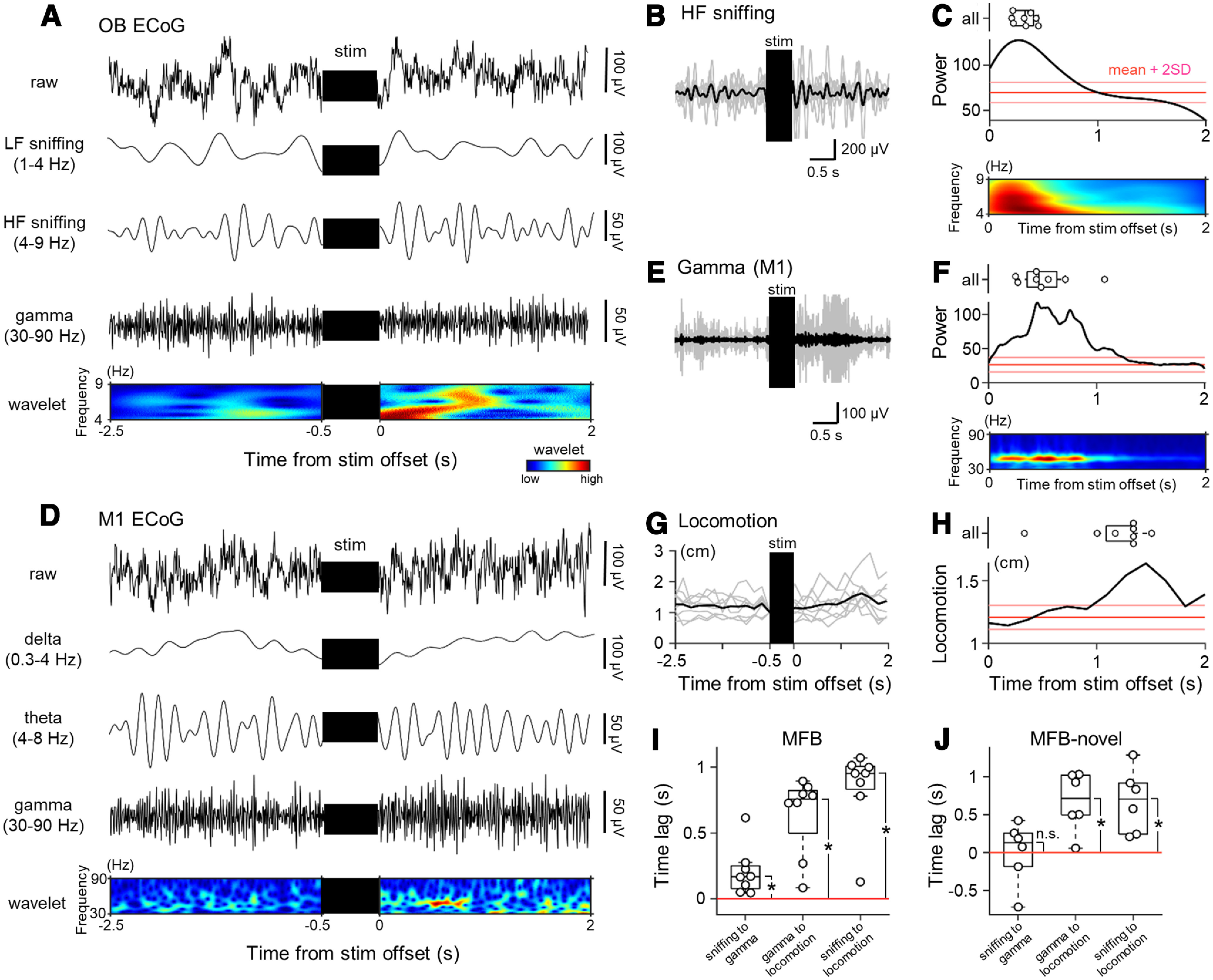
MFB stimulation facilitates sniffing activity and gamma power in the M1, and subsequently induces locomotion. ***A***, Representative raw (top; first) and bandpass-filtered [low-frequency sniffing (1–4 Hz), second; high-frequency sniffing (4–9 Hz), third; gamma (30–90 Hz), fourth] traces of ECoG signals in the OB during the prestimulation and poststimulation periods in the MFB group. The raw trace was convoluted with a Morlet wavelet family and transformed into pseudocolored matrices in the time-frequency domain (fifth). ***B***, Average (black) of the OB ECoG traces bandpass-filtered within 4–9 Hz during the prestimulation and poststimulation periods for rats in the MFB group, superimposed on the traces from each rat (gray). Note that the OB ECoG traces bandpass-filtered within 4–9 Hz indicate high-frequency sniffing activity. ***C***, The raw trace of the OB ECoG signals during the poststimulation period was convoluted with a Morlet wavelet family and transformed into pseudocolored matrices in the time-frequency domain (bottom), yielding the power of the high-frequency sniffing activity based on the wavelet coefficients (middle). The mean (red) and mean ± 2 × SD (pink) of the power during the prestimulation period are shown as thresholds; any suprathreshold values during the poststimulation period are significantly higher than values during the prestimulation period. Note that each of the power trace (middle) and pseudocolored spectrogram (bottom) is not those made from an averaged trace but an average of those made from individual traces. The time that gives the peak power is shown for all rats in the MFB group (top). ***D***, Representative raw [top (first)] and bandpass-filtered [delta (0.3–4 Hz), second; theta (4–8 Hz), third; gamma (30–90 Hz), fourth] traces of ECoG signals in the M1 during the prestimulation and poststimulation periods for rats in the MFB group. The raw trace was convoluted with a Morlet wavelet family and transformed into pseudocolored matrices in the time-frequency domain (fifth). ***E***, Average (black) of the M1 ECoG traces bandpass-filtered in a gamma (30–90 Hz) frequency band during the prestimulation and poststimulation periods for rats in the MFB group, superimposed on the traces from each rat (gray). ***F***, The same as ***C***, but for the gamma power in the M1 ECoG signals. ***G***, The average distance traveled (black) by rats in the MFB group during the prestimulation and poststimulation periods, superimposed on the traces from each rat (gray). ***H***, The expanded trace of the average distance traveled (shown in ***G***) during the poststimulation period (bottom). The time that gives the highest locomotor activity is shown for all rats in the MFB group (top). ***I***, Using the time with the largest values (in ***C***, ***F***, ***H***), the time lag was calculated for all pairs (i.e., “sniffing to gamma,” “gamma to locomotion,” and “sniffing to locomotion”) in the MFB group. ***J***, The same as ***I***, but for the MFB-novel group. The *p* and *t* values were obtained by paired *t* tests (*n *=* *8 and 6 rats in the MFB and MFB-novel groups, respectively). MFB, medial forebrain bundle; ECoG, electrocorticogram; OB, olfactory bulb; M1, primary motor cortex; LF, low-frequency; HF, high-frequency.

**Figure 7.**
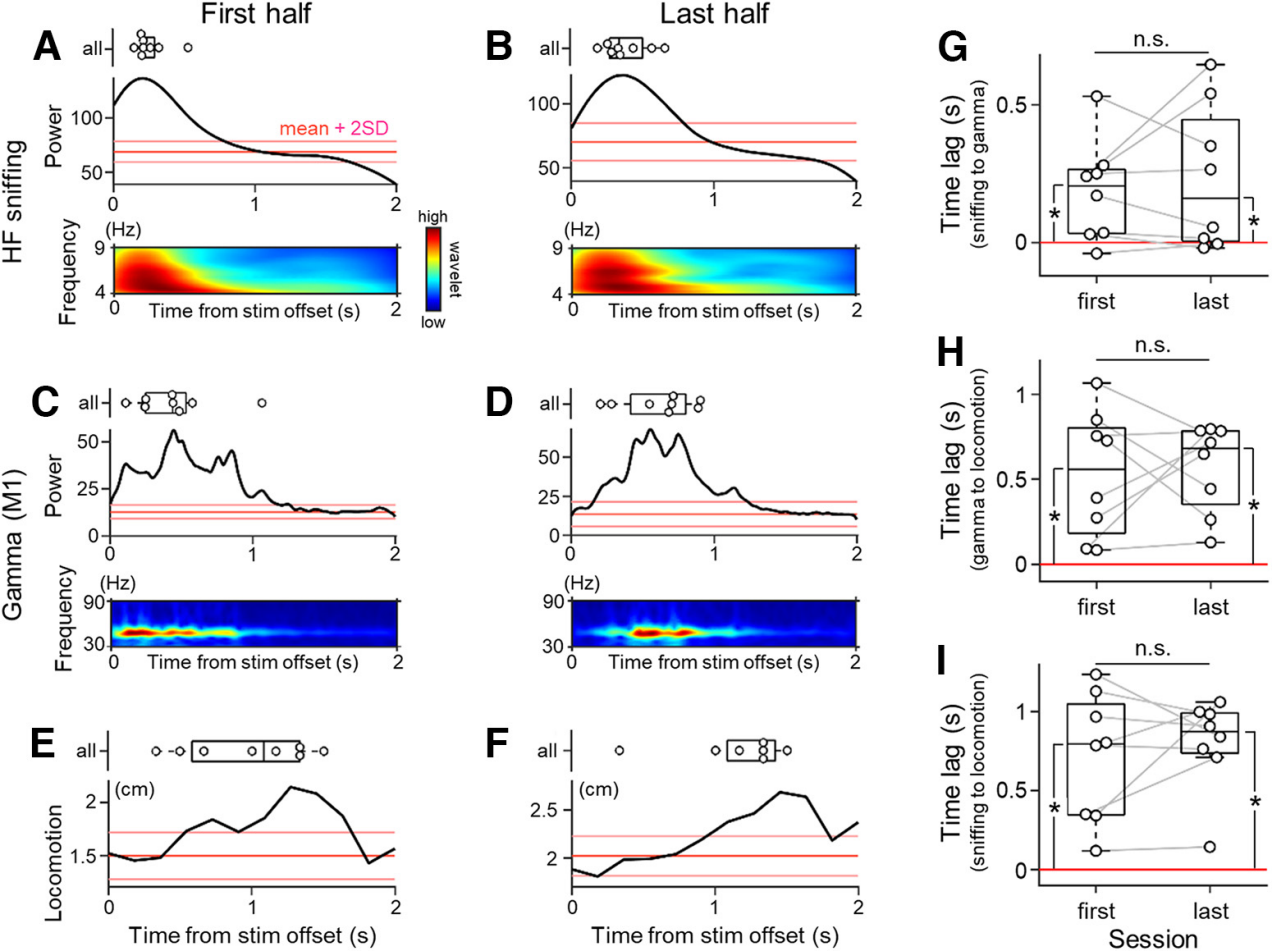
Effects of MFB stimulation on sniffing, M1 gamma power, and locomotion are not different between the first and last halves of the recording sessions. ***A***, For the first half of the whole recording sessions, the raw trace of the OB ECoG signals of rats in the MFB group during the poststimulation period was convoluted with a Morlet wavelet family and transformed into pseudocolored matrices in the time-frequency domain (bottom), yielding the power of the high-frequency sniffing activity based on the wavelet coefficients (middle). The mean (red) and mean ± 2 × SD (pink) of the power during the prestimulation period are shown as thresholds; any suprathreshold values during the poststimulation period are significantly higher than values during the prestimulation period. Note that neither the power trace (middle) nor the pseudocolored spectrogram (bottom) is made from an averaged trace but an average of those made from individual traces. The time that gives the peak power is shown for all rats in the MFB group (top). ***B***, The same as ***A***, but for the last half of the whole recording sessions. ***C***, The same as ***A***, but for the gamma power in the M1 ECoG signals. ***D***, The same as ***C***, but for the last half of the whole recording sessions. ***E***, The average distance traveled by rats in the MFB group during the poststimulation period of the first half of the whole recording sessions (bottom). The time that gives the highest locomotor activity is shown for all rats in the MFB group (top). ***F***, The same as ***E***, but for the last half of the whole recording sessions. ***G***, Time lags from high-frequency sniffing to gamma enhancement in the M1 are not significantly different between the first and last halves of the recording sessions, but both lags are significantly larger than 0 s. Note that the positive value of the lag indicates that high-frequency sniffing precedes gamma enhancement in the M1. ***H***, The same as ***G***, but for the time lags from gamma enhancement to locomotor activity. ***I***, The same as ***G***, but for the time lags from high-frequency sniffing to locomotor activity. The *p* and *t* values were obtained by paired *t* tests (*n *=* *8 rats in the MFB group). HF, high-frequency; M1, primary motor cortex.

To understand the neural oscillatory activity induced by MFB stimulation, the ECoG signals in the OB, M1, and S1 during exploration were converted into the frequency domain data using FFT. Based on the area under the frequency spectra, the ECoG power in a specific frequency band was calculated for the OB [i.e., low-frequency sniffing (1–4 Hz), high-frequency sniffing (4–9 Hz), and gamma (30–90 Hz)] and for the M1 and S1 [i.e., delta (0.3–4 Hz), theta (4–8 Hz), and gamma (30–90 Hz); Figs. 3-7].

The “prestimulation” and “poststimulation” periods were defined as 2 s before and after each stimulation, respectively. To better clarify the precise neural oscillatory activity during both periods, the ECoG signals were further convoluted with a complex Morlet wavelet family (bandwidth parameter, 1.5; center frequency, 2; Figs. 6, 7).

### Code accessibility

Custom-made MATLAB codes for computational analyses are available (Extended Data). To run the codes, Windows was used as the operating system throughout this study. Thus, Windows is recommended to run the MATLAB codes; however, the codes would also run well on either macOS or Linux systems.

10.1523/ENEURO.0521-21.2022.ed Extended Data Custom-made MATLAB codes for computational analyses. Download Extended Data 1, ZIP file.

## Results

### MFB stimulation increases locomotor activity

We implanted a stimulation electrode into the MFB or into a neighboring region of rats. For the rats in the MFB group, we trained them to poke their noses into an active hole for 3 d by delivering electrical stimulation in response to nose-pokes (Fig. 1*A–D*) and confirmed that the number of nose-pokes into the active hole gradually increased day by day in rats in the MFB group [21.00 ± 1.59 (Day 1) vs 129.75 ± 18.47 (Day 2), *p *=* *6.06 × 10^−4^, *t*_(7)_ = 5.88, *n *=* *8 rats, paired *t* test; 21.00 ± 1.59 (Day 1) vs 311.75 ± 13.24 (Day 3), *p *=* *6.14 × 10^−8^, *t*_(7)_ = 23.65, *n *=* *8 rats, paired *t* test; Fig. 1*D*] while that of rats in the sham group in which an unrelated area close to the MFB was stimulated (see Materials and Methods) did not increase [5.00 ± 1.39 (Day 1) vs 4.17 ± 1.22 (Day 2), *p *=* *0.49, *t*_(5)_ = 0.75, *n *=* *6 rats, paired *t* test; 5.00 ± 1.39 (Day 1) vs 4.33 ± 0.99 (Day 3), *p *=* *0.74, *t*_(5)_ =0.35, *n *=* *6 rats, paired *t* test; Fig. 1*D*]. Consistently, the intervals of times between nose-poke events (i.e., interpoke intervals) in the MFB group significantly decreased as the experimental days increased [154.08 ± 27.46 s (Day 1) vs 33.07 ± 12.02 s (Day 2), *p *=* *2.27 × 10^−3^, *t*_(7)_ = 4.68, *n *=* *8 rats, paired *t* test; 154.08 ± 27.46 s (Day 1) vs 19.22 ± 9.00 s (Day 3), *p *=* *4.29 × 10^−3^, *t*_(7)_ = 4.15, *n *=* *8 rats, paired *t* test; Fig. 1*E*]. These behavioral dynamics supported the effectiveness of MFB stimulation in these rats, which we used in the following analyses.

After the rats were fully habituated to an open field (Fig. 2*A*), we allowed them to freely explore the field and simultaneously recorded ECoGs in the S1, M1, and OB for rats in the MFB, sham, and MFB-novel groups during the baseline and stim sessions (Fig. 3*A*,*B*); note that rats in the MFB-novel group had not experienced the apparatus or performed the nose-poke test. During the stim session, electrical stimulation was delivered to rats in both groups at regular intervals (5 s). We tracked and quantified the animal trajectories during both sessions (Fig. 2*B–E*). In the sham group, a rat was likely to prefer a certain location in an open field during both sessions (Fig. 2*D*), suggesting the existence of the rat’s home base (Eilam and Golani, 1989). In contrast, in rats in the MFB group, such a home base disappeared during the stim session compared with the baseline session (Fig. 2*B*). The total distance traveled by rats in the MFB group was significantly longer during the stim session than during the baseline session [0.95 ± 0.15 m/min (baseline) vs 4.89 ± 0.52 m/min (stim), *p *=* *2.21 × 10^−4^, *t*_(7)_ = 6.95, *n *=* *8 rats, paired *t* test; Fig. 2*C*], whereas the total distance traveled by rats in the sham group was not significantly different between the baseline and stim sessions [1.58 ± 0.42 m/min (baseline) vs 1.21 ± 0.22 m/min (stim), *p *=* *0.37, *t*_(5)_ = 0.97, *n *=* *6 rats, paired *t* test; Fig. 2*E*].

To rule out the possibility that the preceding nose-poke performance had an impact on the subsequent home-base behavior and locomotion, we allowed rats in the MFB-novel group to freely explore the open field without performing any nose-poke pretest. In rats in the MFB-novel group, a home base was evident during the baseline session (Fig. 2*F*). Indeed, the home base was still present during the stim session, but compared with the baseline session, the rats visited places other than the original home base more frequently (Fig. 2*F*) and walked a longer distance [1.12 ± 0.32 m/min (baseline) vs 5.00 ± 0.57 m/min (stim), *p *=* *2.71 × 10^−3^, *t*_(5)_ = 5.50, *n *=* *6 rats, paired *t* test; Fig. 2*G*]. These results suggest that MFB stimulation enhances locomotor activity regardless of the preceding nose-poke behavior.

### MFB stimulation facilitates high-frequency sniffing and enhances the gamma power of ECoGs in the M1

To reveal neural activity associated with MFB stimulation-induced locomotion, we analyzed the ECoGs in the OB, M1, and S1 (Fig. 3*A*,*B*). We bandpass-filtered the OB ECoGs at 1–4 Hz, 4–9 Hz, and 30–90 Hz; these frequency bands correspond to low-frequency sniffing, high-frequency sniffing (Kuga et al., 2019), and gamma oscillations, respectively (Bagur et al., 2018). Compared with the baseline, when the MFB was periodically stimulated, we found a significant increase in the power of the OB ECoGs for low-frequency sniffing [2.66 ± 0.94 × 10^3^ μV^2^ (baseline) vs 1.32 ± 0.56 × 10^4^ μV^2^ (stim), *p *=* *0.03, *t*_(7)_ = 2.71, *d *=* *0.96, *n *=* *8 rats, paired *t* test, *p *=* *0.28, *W *=* *0.90, Shapiro–Wilk test; Fig. 3*C*] and for high-frequency sniffing [8.55 ± 2.90 × 10^2^ μV^2^ (baseline) vs 2.72 ± 0.59 × 10^3^ μV^2^ (stim), *p *=* *3.42 × 10^−3^, *t*_(7)_ = 4.33, *d *=* *1.53, *n *=* *8 rats, paired *t* test, *p *=* *0.97, *W *=* *0.98, Shapiro–Wilk test; Fig. 3*C*]. In rats in the sham group, there were no significant differences in the power between the two sessions for low-frequency sniffing [8.97 ± 5.00 × 10^3^ μV^2^ (baseline) vs 1.19 ± 0.55 × 10^4^ μV^2^ (stim), *p *=* *0.08, *t*_(5)_ = 2.15, *n *=* *6 rats, paired *t* test, *p *=* *0.50, *W *=* *0.92, Shapiro–Wilk test; Fig. 3*D*] and for high-frequency sniffing [5.59 ± 3.92 × 10^3^ μV^2^ (baseline) vs 2.44 ± 0.99 × 10^3^ μV^2^ (stim), *p *=* *0.44, *W *=* *15, *n *=* *6 rats, Wilcoxon signed-rank test; *p *=* *4.65 × 10^−2^, *W *=* *0.79, Shapiro–Wilk test; Fig. 3*D*]. In addition, the gamma (30–90 Hz) power of the OB ECoGs was not significantly different between the two sessions for rats in either the MFB group [5.30 ± 2.40 × 10^3^ μV^2^ (baseline) vs 2.67 ± 1.13 × 10^3^ μV^2^ (stim), *p *=* *0.12, *t*_(7)_ = 1.77, *n *=* *8 rats, paired *t* test, *p *=* *0.66, *W *=* *0.95, Shapiro–Wilk test; Fig. 3*C*] or the sham group [1.46 ± 0.71 × 10^3^ μV^2^ (baseline) vs 1.28 ± 0.28 × 10^3^ μV^2^ (stim), *p *=* *0.59, *t*_(5)_ = 0.57, *n *=* *6 rats, paired *t* test, *p *=* *0.61, *W *=* *0.93, Shapiro–Wilk test; Fig. 3*D*]. Based on the effect size (Cohen, 1988; Sawilowsky, 2009), we assumed that MFB stimulation had a larger effect on high-frequency sniffing power than on low-frequency sniffing power.

Next, we bandpass-filtered the M1 ECoGs within a specific frequency range [i.e., 0.3–4 Hz (delta), 4–8 Hz (theta), and 30–90 Hz (gamma)]. In rats in the MFB group, the bandpass-filtered M1 ECoG power during the stim session was significantly larger than that during the baseline session for delta [1.57 ± 0.68 × 10^3^ μV^2^ (baseline) vs 1.37 ± 0.91 × 10^5^ μV^2^ (stim), *p *=* *0.02, *t*_(7)_ = 3.10, *n *=* *8 rats, paired *t* test, *p *=* *0.42, *W *=* *0.92, Shapiro–Wilk test; Fig. 3*E*], theta [2.37 ± 1.82 × 10^3^ μV^2^ (baseline) vs 3.32 ± 2.78 × 10^4^ μV^2^ (stim), *p *=* *0.02, *t*_(7)_ = 3.16, *n *=* *8 rats, paired *t* test, *p *=* *0.22, *W *=* *0.89, Shapiro–Wilk test; Fig. 3*E*], and gamma frequency bands [9.26 ± 5.66 × 10^2^ μV^2^ (baseline) vs 4.07 ± 1.69 × 10^3^ μV^2^ (stim), *p *=* *0.01, *t*_(7)_ = 3.22, *n *=* *8 rats, paired *t* test; *p *=* *0.43, *W *= 0.93, Shapiro–Wilk test; Fig. 3*E*]. However, there were no significant differences in the power in the M1 ECoGs between the two sessions in rats in the sham group for any frequency band [delta, 3.10 ± 2.36 × 10^5^ μV^2^ (baseline) vs 3.47 ± 2.63 × 10^5^ μV^2^ (stim), *p *=* *0.07, *t*_(5)_ = 2.34, *n *=* *6 rats, paired *t* test, *p *=* *0.72, *W *=* *0.95, Shapiro–Wilk test; theta, 1.44 ± 1.29 × 10^5^ μV^2^ (baseline) vs 7.20 ± 4.62 × 10^4^ μV^2^ (stim), *p *=* *0.14, *t*_(5)_ = 1.73, *n *=* *6 rats, paired *t* test, *p *=* *0.93, *W *=* *0.98, Shapiro–Wilk test; gamma, 1.09 ± 0.69 × 10^4^ μV^2^ (baseline) vs 1.64 ± 1.07 × 10^4^ μV^2^ (stim), *p *=* *0.27, *t*_(5)_ = 1.25, *n *=* *6 rats, paired *t* test, *p *=* *0.47, *W *=* *0.91, Shapiro–Wilk test; Fig. 3*F*].

To exclude the possibility of EMGs contaminating the M1 ECoGs (and hence gamma power enhancement in the M1), we analyzed the S1 ECoGs in the same manner as we evaluated the M1 ECoGs (Fig. 4). In contrast to the M1, the bandpass-filtered power of the S1 ECoGs was not significantly different between the two sessions for rats either in the MFB group [delta, 2.22 ± 1.45 × 10^4^ μV^2^ (baseline) vs 1.78 ± 0.94 × 10^5^ μV^2^ (stim), *p *=* *0.21, *t*_(7)_ = 1.39, *n *=* *8 rats, paired *t* test, *p *=* *0.69, *W *=* *0.95, Shapiro–Wilk test; theta, 3.56 ± 1.51 × 10^3^ μV^2^ (baseline) vs 1.78 ± 0.94 × 10^5^ μV^2^ (stim), *p *=* *0.10, *t*_(7)_ = 1.90, *n *=* *8 rats, paired *t* test, *p *=* *0.13, *W *=* *0.87, Shapiro–Wilk test; gamma, 1.03 ± 0.97 × 10^4^ μV^2^ (baseline) vs 2.51 ± 1.85 × 10^4^ μV^2^ (stim), *p *=* *0.09, *t*_(7)_ = 1.97, *n *=* *8 rats, paired *t* test; *p *=* *0.35, *W *=* *0.92, Shapiro–Wilk test; Fig. 4*A*] or in the sham group [delta, 3.02 ± 2.71 × 10^5^ μV^2^ (baseline) vs 7.36 ± 3.16 × 10^5^ μV^2^ (stim), *p *=* *0.10, *t*_(5)_ = 2.03, *n *=* *6 rats, paired *t* test, *p *=* *0.91, *W *=* *0.97, Shapiro–Wilk test; theta, 1.24 ± 1.20 × 10^5^ μV^2^ (baseline) vs 2.48 ± 1.38 × 10^5^ μV^2^ (stim), *p *=* *0.14, *t*_(5)_ = 1.74, *n *=* *6 rats, paired *t* test, *p *=* *0.81, *W *=* *0.96, Shapiro–Wilk test; gamma, 7.56 ± 6.84 × 10^3^ μV^2^ (baseline) vs 2.29 ± 1.17 × 10^4^ μV^2^ (stim), *p *=* *0.17, *t*_(5)_ = 1.58, *n *=* *6 rats, paired *t* test, *p *=* *0.80, *W *=* *0.96, Shapiro–Wilk test; Fig. 4*B*].

To further tease out the effects of the preceding nose-poke behavior on the subsequent ECoG data, we also analyzed ECoGs in the OB, M1, and S1 in rats in the MFB-novel group. Consistent with the outcomes observed for rats in the MFB group, for the OB ECoGs, we found that the high-frequency sniffing component was significantly higher during the stim session than during the baseline session [1.44 ± 0.39 × 10^3^ μV^2^ (baseline) vs 3.34 ± 0.47 × 10^3^ μV^2^ (stim), *p *=* *0.03, *W *=* *21, *n *=* *6 rats, Wilcoxon signed-rank test, *p *=* *0.03, *W *=* *0.77, Shapiro–Wilk test; Fig. 5*A*]; note that the low-frequency sniffing component was also increased during the stim session [5.80 ± 1.83 × 10^3^ μV^2^ (baseline) vs 1.78 ± 0.50 × 10^4^ μV^2^ (stim), *p *=* *0.03, W = 21, *n *=* *6 rats, Wilcoxon signed-rank test, *p *=* *4.70 × 10^−2^, W = 0.79, Shapiro–Wilk test; Fig. 5*A*]. There was no significant difference in the gamma frequency component between the two sessions [1.12 ± 0.42 × 10^3^ μV^2^ (baseline) vs 5.56 ± 4.01 × 10^3^ μV^2^ (stim), *p *=* *0.07, *t*_(5)_ = 2.32, *n *=* *6 rats, paired *t* test, *p *=* *0.48, *W *=* *0.92, Shapiro–Wilk test; Fig. 5*A*]. Additionally, the gamma power in the M1 ECoGs was significantly enhanced during the stim session [8.16 ± 3.82 × 10^2^ μV^2^ (baseline) vs 6.41 ± 5.28 × 10^3^ μV^2^ (stim), *p *=* *0.03, *W *=* *21, *n *=* *6 rats, Wilcoxon signed-rank test, *p *=* *4.77 × 10^−3^, *W *=* *0.65, Shapiro–Wilk test; Fig. 5*B*], whereas neither the delta nor theta power was enhanced [delta, 5.00 ± 2.67 × 10^3^ μV^2^ (baseline) vs 7.24 ± 3.80 × 10^3^ μV^2^ (stim), *p *=* *0.09, *t*_(5)_ = 2.10, *n *=* *6 rats, paired *t* test, *p *=* *0.34, *W *=* *0.89, Shapiro–Wilk test; theta, 3.32 ± 1.92 × 10^3^ μV^2^ (baseline) vs 3.45 ± 1.53 × 10^3^ μV^2^ (stim), *p *=* *0.10, *t*_(5)_ = 2.03, *n *=* *6 rats, paired *t* test, *p *=* *0.85, *W *=* *0.96, Shapiro–Wilk test; Fig. 5*B*]. As was the case with rats in the MFB group, we failed to find any significant differences in the delta or gamma frequency component of the S1 ECoGs between the two sessions [delta, 5.04 ± 2.45 × 10^3^ μV^2^ (baseline) vs 1.72 ± 0.96 × 10^4^ μV^2^ (stim), *p *=* *0.09, *t*_(5)_ = 2.10, *n *=* *6 rats, paired *t* test, *p *=* *0.75, *W *=* *0.95, Shapiro–Wilk test; gamma, 9.40 ± 4.39 × 10^2^ μV^2^ (baseline) vs 2.09 ± 1.40 × 10^4^ μV^2^ (stim), *p *=* *0.11, *t*_(5)_ = 1.97, *n *=* *6 rats, paired *t* test, *p *=* *0.07, *W *=* *0.81, Shapiro–Wilk test; Fig. 5*C*], whereas the theta frequency component was increased [4.04 ± 1.70 × 10^3^ μV^2^ (baseline) vs 1.26 ± 0.75 × 10^4^ μV^2^ (stim), *p *=* *0.03, *W *=* *21, *n *=* *6 rats, Wilcoxon signed-rank test, *p *=* *0.03, *W *=* *0.77, Shapiro–Wilk test; Fig. 5*C*].

Altogether, these results suggest that repeated MFB stimulation acutely affected the high-frequency sniffing component in the OB ECoGs and the gamma oscillations in the M1.

### MFB stimulation induces sniffing and facilitates motor cortical gamma oscillations preceding locomotion

Since MFB stimulation facilitated gamma oscillations in the M1, induced locomotion and provoked high-frequency sniffing activity, we investigated the temporal relationship among these phenomena. For the MFB group, we bandpass-filtered the original ECoGs in the OB between 4 and 9 Hz (Fig. 6*A*,*B*) and estimated the time-varying high-frequency sniffing components during the poststimulation period based on spectral analysis (Fig. 6*C*). The sniffing component peaked immediately after the stimulation terminated and declined following that peak.

We then investigated how MFB stimulation modulated motor cortical neural activity because we found enhanced gamma power in the M1 ECoGs but not in the S1 ECoGs (Figs. 3-5). We bandpass-filtered the M1 ECoGs within three frequency ranges (i.e., delta, theta, and gamma) and convoluted the raw signals using a complex Morlet wavelet family (Fig. 6*D*), which demonstrated sustained enhancement of gamma power in the M1 after MFB stimulation (Fig. 6*D*,*E*). In the same manner as the sniffing components (Fig. 6*C*), we calculated the time-varying changes in the gamma component in the M1 ECoGs (Fig. 6*F*). We further quantified locomotor activity during the prestimulation and poststimulation periods (Fig. 6*G*,*H*) and revealed that the gamma power peaked before the locomotor activity reached the maximum level (Fig. 6*E–H*).

We calculated a time lag based on the time when the sniffing, gamma power, and locomotor activity reached the maximum. For the MFB group, the time lag from sniffing activity to gamma power enhancement was significantly larger than 0 s (0.30 ± 0.08 s, *p *=* *1.98 × 10^−3^, *t*_(7)_ = 4.80, *n *=* *8 rats, one-sample *t* test vs 0 s; Fig. 6*I*), whereas the time lag from sniffing to gamma was not significantly different from 0 s for the MFB-novel group (0.01 ± 0.17 s, *p *=* *0.97, *t*_(5)_ = 0.04, *n *=* *6 rats, one-sample *t* test vs 0 s; Fig. 6*J*). These results suggest that the facilitation of sniffing activity and the enhancement of gamma oscillatory activity in the M1 partially overlap during the period following MFB stimulation; the sniffing facilitation does not necessarily precede the motor cortical gamma enhancement. In contrast, the time lag from the gamma enhancement to the peak of locomotor activity was significantly larger than 0 s for the MFB group (0.49 ± 0.06 s, *p *=* *1.98 × 10^−3^, *t*_(7)_ = 4.80, *n *=* *8 rats, one-sample *t* test vs 0 s; Fig. 6*I*) and the MFB-novel group (0.67 ± 0.16 s, *p *=* *8.18 × 10^−3^, *t*_(5)_ = 4.24, *n *=* *6 rats, one-sample *t* test vs 0 s; Fig. 6*J*). Moreover, the time lag from the sniffing to the locomotion was also significantly larger than 0 s for the MFB group (0.85 ± 0.11 s, *p *=* *9.91 × 10^−5^, *t*_(7)_ = 7.90, *n *=* *8 rats, one-sample *t* test vs 0 s; Fig. 6*I*) and the MFB-novel group (0.68 ± 0.17 s, *p *=* *1.06 × 10^−2^, *t*_(5)_ = 3.97, *n *=* *6 rats, one-sample *t* test vs 0 s; Fig. 6*J*). Taken together, the MFB stimulation-induced oscillatory activity in the OB and M1 was followed by the locomotor activity in all groups.

To clarify how the effect of MFB stimulation was robust throughout the sessions, we divided the sessions into the first and last halves and investigated the temporal relationship among the sniffing activity, gamma oscillations, and locomotion in each half by estimating the time-varying high-frequency sniffing components (Fig. 7*A*,*B*), gamma components in the M1 ECoGs (Fig. 7*C*,*D*) and locomotor activity (Fig. 7*E*,*F*) during the poststimulation period. We quantified the time lags (1) from high-frequency sniffing activity to the point of gamma enhancement, (2) from gamma enhancement to locomotion, and (3) from sniffing to locomotion (Fig. 7*G–I*). There were no significant differences in the time lag (1) from sniffing to the point of gamma enhancement [0.26 ± 0.04 s (first) vs 0.39 ± 0.06 s (last), *p *=* *0.54, *t*_(7)_ = 0.63, *n *=* *8 rats, paired *t* test; Fig. 7*G*], (2) from gamma enhancement to locomotion [0.45 ± 0.10 s (first) vs 0.62 ± 0.09 s (last), *p *=* *0.79, *t*_(7)_ = 0.28, *n *=* *8 rats, paired *t* test; Fig. 7*H*], or (3) from sniffing to locomotion [0.98 ± 0.15 s (first) vs 1.19 ± 0.14 s (last), *p *=* *0.50, *t*_(7)_ = 0.70, *n *=* *8 rats, paired *t* test; Fig. 7*I*] between the first and last halves of the recording sessions. (1) The time lag from sniffing to gamma enhancement was significantly higher than 0 s for each of the first and last halves of the sessions (first, *p *=* *0.02, *t*_(7)_ = 2.90, *n *=* *8 rats, one-sample *t* test vs 0 s; last, *p *=* *0.04, *t*_(7)_ = 2.49, *n *=* *8 rats, one-sample *t* test vs 0 s; Fig. 7*G*). Similarly, (2) the time lags from gamma enhancement to locomotion were significantly above 0 s (first, *p *=* *4.84 × 10^−3^, *t*_(7)_ = 4.05, *n *=* *8 rats, one-sample *t* test vs 0 s; last, *p *=* *4.52 × 10^−4^, *t*_(7)_ = 6.18, *n *=* *8 rats, one-sample *t* test vs 0 s; Fig. 7*H*) and (3) the time lags from sniffing to locomotion significantly exceeded 0 s (first, *p *=* *1.56 × 10^−3^, *t*_(7)_ = 7.90, *n *=* *8 rats, one-sample *t* test vs 0 s; last, *p *=* *1.10 × 10^−4^, *t*_(7)_ = 7.77, *n *=* *8 rats, one-sample *t* test vs 0 s; Fig. 7*I*). To recap, these results suggested that the acute effects of MFB stimulation on sequential modification of neural activity and behavior were robust.

## Discussion

In this study, we found that electrical stimulation of the rat MFB increased exploratory behavior, sniffing activity, and extracellular gamma oscillatory power in the M1. Moreover, the time-series analysis confirmed that MFB stimulation enhanced sniffing activity and gamma power in the M1, and subsequently induced locomotion.

As a neural reward, MFB stimulation motivates animals so powerfully that their behaviors are dynamically modified (Talwar et al., 2002). Rats learned to exhibit instrumental (e.g., nose-poking and lever-pressing) behavior faithfully and quickly (Fig. 1*D*). Additionally, the locomotor activity of the rats in the MFB group was enhanced (Fig. 2*B*,*C*); this activity may be mediated by dopamine D1 receptors (Tran et al., 2005). Moreover, it is well known that rats alternately run and stop when they are placed in an environment, but they are likely to stop at one or two specific places, defined as their home base (Eilam and Golani, 1989); home bases can be modulated to some extent by salient stimuli and environmental geometry (Thompson et al., 2018). Here, the trajectory of a rat in the sham group during the stim session confirmed that the rat frequently crossed a specific location (Fig. 2*D*), which can be regarded as the rat’s home base. Home bases were also observed in rats in the sham, MFB, and MFB-novel groups during the baseline session (Fig. 2*B*,*D*,*F*). However, rats in the MFB and MFB-novel groups during the stim session explored not only around their specific home bases but also around every corner and beside every wall in the open field, suggesting that acute MFB stimulation diminished home base behavior. We assumed that reward-seeking responses evoked by the preceding MFB stimulation resulted in the disappearance of the home base behavior (Margules and Olds, 1962; Wise, 2005), regardless of preexposure to the conditioning apparatus with nose-poke holes.

We also scrutinized how this behavioral modification was associated with neural activity in the OB and M1 (Figs. 3, 5). Regarding OB activity, high-frequency sniffing is often observed when animals are motivated to explore an external environment (Wesson et al., 2008; Kuga et al., 2019) and may play a role in the acquisition of olfactory information to guide their ongoing behavior (Kepecs et al., 2006; Ranade et al., 2013; Kleinfeld et al., 2016). Consistent with a previous study on sniffing responses based on thermal changes in the rat nasal cavity (Waranch and Terman, 1975), we observed intense high-frequency sniffing activity immediately after MFB stimulation (Fig. 6*A–C*). Since there were no odor cues in our experimental setup as a matter of course, we considered that rats were driven to “virtually” incorporate sensory information into themselves to search for the origin of rewards; this approach contributed to reward-seeking behavior and a gradual increase in subsequent locomotion (Fig. 6*G*,*H*). Moreover, the sniffing activity of mice is increased in anticipation of future reward delivery (Wesson et al., 2008). Thus, enhanced sniffing activity preceding locomotion appears to signify reward-seeking and reward-anticipating behavior.

In addition to sniffing activity, we found that MFB stimulation enhanced the power of delta, theta, and gamma oscillations in M1 ECoGs of well-trained rats in the MFB group (Fig. 3); however, we should also hasten to add that the only gamma power was increased in completely naive (i.e., preexposure-free) rats in the MFB-novel group (Fig. 5). Regarding the mechanism underlying the MFB stimulation-induced enhancement of M1 gamma oscillations, we considered neural projections to the M1 via the MFB, although we cannot completely exclude the possibility that sniffing activity directly affected M1 ECoGs. The MFB is considered to connect several brain areas, including the VTA, lateral and medial hypothalamus, and ventral striatum (Gálvez et al., 2015). Among these brain areas, dopaminergic neurons in the VTA project to the M1 in rats (Lindvall et al., 1978; Luft and Schwarz, 2009; Hosp et al., 2011) and humans (Hosp et al., 2019). Intrinsic properties and synaptic transmission of M1 parvalbumin-positive interneurons are modulated by dopaminergic signals via dopamine D2 receptors (Cousineau et al., 2020; Duan et al., 2020); of note, VTA neurons innervate parvalbumin-positive interneurons in the M1 (Duan et al., 2020). Fast-spiking activity of parvalbumin-positive neurons is believed to produce gamma oscillations via synchronized inhibitory synaptic currents in cortical pyramidal cells (Buzsáki and Wang, 2012). Thus, we speculate that the MFB stimulation-induced enhancement of M1 gamma oscillations is mediated by dopaminergic signals sent from the VTA to the M1.

Despite the MFB stimulation-induced enhancement of M1 gamma oscillations, it is surprising, to some extent, that we did not observe either enhancement or impairment of oscillatory power in the S1 ECoGs in rats in the MFB or MFB-novel group because sniffing and whisking activities are tightly coupled with each other and both activities are involved in reward-seeking and reward-anticipating behaviors. Although we did not provide experimental proof, we speculate that the possible mechanism underlying the lack of an effect of MFB stimulation on the S1 ECoGs is related to the release of acetylcholine. Previous studies demonstrated that higher concentrations of acetylcholine are released in the S1 than the M1 in the nocturnal phase (Jiménez-Capdeville and Dykes, 1996) and that cholinergic neuronal activity is associated with desynchronized extracellular oscillations (Blake and Boccia, 2016). Therefore, compared with the M1, more desynchronization of neural activity in the S1 may have brought about more variability in the oscillatory change and overwhelmed sniffing/whisking-induced neural activity.

Compared with the completely awake animals used here, a previous study measured single-cell unit activity in the thalamus and brainstem and electroencephalograms (EEGs) in the frontal and occipital cortices of anesthetized rats simultaneously with MFB stimulation (Rolls, 1971). This study indicated that, based on desynchronization of the cortical EEGs, the anesthetized rats were forced to be somewhat awake when receiving MFB stimulation at least at the firing and oscillatory activity levels (Rolls, 1971). The observation of attenuated EEG signals appears to contradict our current findings that the multiple oscillatory (i.e., delta, theta, and gamma) powers in the M1 ECoGs of the MFB group were enhanced by MFB stimulation (Fig. 3*E*). However, we assume that this contradiction originates from the fact that MFB stimulation excited only a subpopulation of neurons in anesthetized rats (Rolls, 1971). This previous study divided the MFB stimulation-responsive firing units into three types: antidromically driven (i.e., directly excited) brainstem units, monosynaptically driven brainstem units, and multisynaptically driven units in the brainstem and thalamus (Rolls, 1971). Importantly, MFB stimulation was considered to antidromically excite brainstem neurons and further excite neurons downstream of the “antidromically excited” neurons via synapses. The antidromically driven units did not exhibit firing rates that correlated with the real awake state, whereas some of the monosynaptically driven units and all multisynaptically units had firing rates that resembled firing rates under arousal (Rolls, 1971). In this sense, the completely waking state in this study and the MFB stimulation-induced pseudoarousal under anesthesia by urethane and equithesin are totally different (Rolls, 1971; Batzri-Izraeli et al., 1992). Hence, MFB stimulation-induced enhancement of a wide range of the power of the M1 ECoGs (of rats in the MFB group) is an awake state-specific phenomenon.

Although we demonstrated that the extracellular gamma oscillations in the M1 were facilitated by MFB stimulation, how MFB stimulation affects M1 neural activity at the synaptic level and contributes to behavioral functions remains to be fully elucidated. In this light, the previous histologic evidence provides insights that could address the question. Expression of c-Fos protein, an immediate early gene (i.e., *c-fos*) product, is induced in the M1 by dopamine release on electrical stimulation in the VTA (Hosp et al., 2011). Dopamine is also involved in long-term synaptic plasticity in the M1 (Rioult-Pedotti et al., 2015). These studies suggested that synaptic plasticity in the M1 may be induced when rewarding dopaminergic signals are sent from the VTA to the M1. Motor learning is accompanied by synaptic plasticity in the M1 (Rioult-Pedotti et al., 1998, 2000, 2015); thus, dopaminergic signals should contribute to motor learning (Molina-Luna et al., 2009).

In addition to the possible synaptic plasticity in the M1 induced by MFB stimulation, we assume that motor cortical gamma oscillations potentially impact learning. Indeed, we have not empirically demonstrated whether or how motor cortical gamma oscillations induced by MFB stimulation serve to promote motor learning; however, a previous study using rats showed that gamma oscillations in the M1 were dominant during a lever-pressing task associated with rewards (Igarashi et al., 2013). A recent study suggested that gamma oscillations in the rat M1 regulate motor learning (Otsuka and Kawaguchi, 2021). Taken together, it is plausible that dopaminergic signals elicited by MFB stimulation facilitate motor learning via gamma oscillations and synaptic plasticity in the M1; this relation could be further elucidated by behavioral electrophysiology with an operant task.

## Synthesis

Reviewing Editor: Christina Zelano, Northwestern University - Chicago Decisions are customarily a result of the Reviewing Editor and the peer reviewers coming together and discussing their recommendations until a consensus is reached. When revisions are invited, a fact-based synthesis statement explaining their decision and outlining what is needed to prepare a revision will be listed below. The following reviewer(s) agreed to reveal their identity: Andrew Moberly.

The authors have made a thorough effort to address concerns raised by both reviewers and we are largely satisfied by their responses. Here, they demonstrate that rats will increase rates of nose-poking if this action is coupled to MFB stimulation, MFB stimulation has effects on OB and M1 oscillatory activity (but not S1) and locomotor activity.

The initial findings were largely recapitulated in a new experimental group of 6 rats that received MFB stimulation for the first time in a novel arena, although differences in oscillatory activity in M1 between the naïve and trained mice could be related to the hundreds of MFB stimulations delivered over the course of 3 training days.

Also, it appears that the wavelet and time lag analyses were not provided for this experimental group. We are not suggesting that the authors need to re-create figures 6-7 with these animals, but since these data were collected, some comment on how they compare with the original data set from the 8 rats that underwent nose-poke training should be appropriate.

The authors seem to have taken a strong position on the ordering of the stim-evoked physiological and behavioral phenomena (first changes to OB activity, then increases in M1 gamma then locomotion). Is there a strong motivation to emphasize this? The alternative is that the changes in OB an M1 oscillatory power largely overlap in time following MFB stimulation. One gets the sense from the distributions of peaks and time-frequency plots in Fig6c-f and Fig7a-d that this is the case. If these distributions are directly compared (as opposed to subtracted and compared with 0) are they still significantly different?

Additional Minor point- it looks like Figure 2 is missing a figure legend for panel F.
